# Chain organization of human interphase chromosome determines the spatiotemporal dynamics of chromatin loci

**DOI:** 10.1371/journal.pcbi.1006617

**Published:** 2018-12-03

**Authors:** Lei Liu, Guang Shi, D. Thirumalai, Changbong Hyeon

**Affiliations:** 1 Korea Institute for Advanced Study, Seoul, Korea; 2 Biophysics Program, Institute for Physical Science and Technology, University of Maryland, College Park, MD, USA; 3 Department of Chemistry, The University of Texas at Austin, Austin, TX, USA; Ottawa University, CANADA

## Abstract

We investigate spatiotemporal dynamics of human interphase chromosomes by employing a heteropolymer model that incorporates the information of human chromosomes inferred from Hi-C data. Despite considerable heterogeneities in the chromosome structures generated from our model, chromatins are organized into crumpled globules with space-filling (SF) statistics characterized by a single universal scaling exponent (*ν* = 1/3), and this exponent alone can offer a quantitative account of experimentally observed, many different features of chromosome dynamics. The local chromosome structures, whose scale corresponds to that of topologically associated domains (∼ 0.1 − 1 Mb), display dynamics with a fast relaxation time (≲ 1 − 10 sec); in contrast, the long-range spatial reorganization of the entire chromatin ($≳\;O(10^{2})$ Mb) occurs on a much slower time scale (≳ hour), providing the dynamic basis of cell-to-cell variability and glass-like behavior of chromosomes. Biological activities, modeled using stronger isotropic white noises added to active loci, accelerate the relaxation dynamics of chromatin domains associated with the low frequency modes and induce phase segregation between the active and inactive loci. Surprisingly, however, they do not significantly change the dynamics at local scales from those obtained under passive conditions. Our study underscores the role of chain organization of chromosome in determining the spatiotemporal dynamics of chromatin loci.

## Author summary

Chromosomes are giant chain molecules made of hundreds of megabase-long DNA intercalated with proteins. Structure and dynamics of interphase chromatin in space and time hold the key to understanding the cell type-dependent gene regulation. In this study, we establish that the crumpled and space-filling (SF) organization of chromatin fiber in the chromosome territory, characterized by a single scaling exponent, is sufficient to explain the complex spatiotemporal hierarchy in chromatin dynamics as well as the subdiffusive motion of the chromatin loci. While seemingly a daunting problem at a first glance, our study shows that relatively simple principles, rooted in polymer physics, can be used to grasp the essence of dynamical properties of the interphase chromatin.

## Introduction

The three dimensional (3D) structures of chromosome vary with the developmental stage [1] and cell types, which implies that knowledge of chromosome structure and dynamics is key to understanding their link to gene regulation [2]. A well-designed chromosome structure can facilitate long range transcriptional regulation by keeping two distal genomic loci of enhancer and promoter in proximity [3–5]. Hierarchical organization of chromosomes are inferred from the patterns of Hi-C maps which measure mean contact frequencies of cross-linking between DNA segments based on an ensemble of millions of fixed cells. Chromosomes at ∼ 5 Mb resolution are partitioned into alternating A and B type compartments that are enriched with active and inactive loci, respectively [6]. Hi-C data at submegabase resolution offer glimpses into the structure of TADs (topologically associated domains), the functional building blocks of interphase chromosome [7, 8]. Genome-wide Hi-C maps at even higher resolution of ∼O(10) Kb suggests that each type of compartment is associated with distinct epigenetic pattern, further segregating into six sub-compartments [9]. In addition, fluorescence images visualizing real-time chromatin dynamics *in vivo* [10–13] allow us to decipher the link between structure, dynamics, and function [14–16].

Along with the above-mentioned knowledge from measurements, extensive effort has also been made in developing polymer models for the 3D organization of chromosomes [17–23] and their dynamics [24–30]. For example, ‘strings and binders switch (SBS)’ model, originally proposed to explain many generic behaviors of chromatin within living cells [19], has recently been further extended to explore the hierarchical chromosome structures [31] and the effects of structural variants on chromatin architecture [23]. More recently, chromatins have been modeled as a block polymer condensed by bivalent or multivalent binding factors, mimicking the binding of transcription factors; while mainly focusing on structural properties, the model has shown how an extended chain is collapsed, and discuss how domains are formed [32]. The loop extrusion polymer model [20, 21], based on the knowledge of the convergent orientation of the CTCF-binding motifs, has been used to explain the formation of TADs and predict the contact maps of edited genomes upon deletion of CTCF-binding sites [20, 21]. There is also a growing trend to integrate the data from Hi-C, fluorescence *in situ* hybridization (FISH), and epigenetic states into a block copolymer-type model in order to more realistically design 3D chromosome structures and their role in biological function [33–39]. However, homopolymer models with geometrical and topological constraints alone [6, 28, 40–43] may suffice in capturing some of the physical bases of chromosome organization.

The primary aim of this study is to elucidate the principles underlying the intra-chromosomal *dynamics* in space and time, which has been underappreciated in theoretical and computational studies than the problem of inferring chromosome structure from Hi-C data. A heterogeneous population of conformational ensemble of chromosomes was generated by using one of the recently proposed heteropolymer models,—Minimal Chromatin Model (MiChroM)—whose parameters were trained for the Hi-C data of chromosome 10 (Chr10) from human B-lymphoblastoid cell [22]. To study dynamics of chromosomes we modified the original MiChroM, which is partially self-avoiding with an energetic penalty for each crossing, by imposing a strict self-avoidance constraint and performed Brownian dynamics simulations. Discussing their dynamic properties using various correlation functions, we show that the basic features of the chromatin *dynamics* reported in the recent experiments [44, 45] can be explained quantitatively by the crumpled, hierarchical, territorial, summarized as space-filling organization of chromatin chain. Finally, by incorporating active noises onto *active* loci, we investigate the contribution of activity to the dynamic properties of the interphase chromatin.

## Results

### Heteropolymer model for chromosome

**Fig 1:**
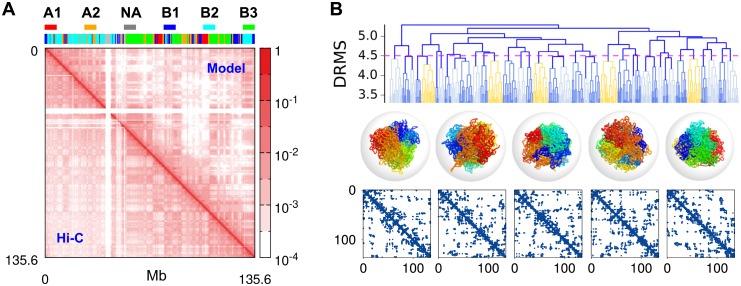
Conformational ensemble of chromosome 10 of human B-lymphoblastoid cells generated from simulations. (A) The contact frequency map from the ensemble of structures generated using MiChroM (upper right corner) generates the overall checkerboard pattern of Hi-C map (lower left corner). The 6 subcompartment types assigned to chromosome loci are depicted on the top. (B) The dendrogram represents the outcome of hierarchical clustering of the ensemble of structures obtained from conformational sampling. Each terminal branch at DRMS = 3.3 *a* represents the ensemble of structures that can be clustered with the condition of DRMS < 3.3 *a*. The distance (DRMS) between the two distinct structures *k* and *l* is given by $D_{k,l}$ (Eq 1), and the distance between two clusters *K* and *L* is defined as the maximum distance between two conformations, each belonging to the two clusters, i.e., $\max_{k\in K,l\in L}\left\{D_{k,l}\right\}$. Among the clusters whose inter-cluster distance is smaller than $D_{c}=4.5a$, the centroid structures (*k*_*c*_ ∈ *K*), which minimize $\sum_{k_{c},k\in K}D_{k_{c},k}$, are depicted in rainbow coloring scheme. As suggested by the contact map of each chromosome structure shown at the bottom, the centroid structure of each cluster is distinct from each other. We have selected these five structures as the initial conformations for generating trajectories for dynamic simulations of chromosomes.

We use MiChroM [22], a 3D coarse-grained heteropolymer model, to study chromosome dynamics at genomic scales greater than 50 Kb. In the model one of the 6 subcompartment types (B3, B2, B1, NA, A1, and A2) (see the color barcode above Fig 1A), determined based on the correlation between the distinct patterns of interchromosomal contacts and epigenetic information [9], is assigned to each monomer representing 50 Kb of DNA segment. In the Hi-C map, potential binding sites for CTCF [20] display higher contact frequencies than their local background. The interactions for chromosome are implemented in the model in terms of the energy potentials of (i) a homopolymer, (ii) monomer type dependent interactions, (iii) attractions between loop sites, and (iv) genomic distance-dependent condensation energies (See SI for details). We note that due to intra-chromosomal interactions, the effect of the confining sphere used in this model, which gives rise to a volume fraction of 10% (*ϕ* = 0.1), is not significant enough to alter the chromosome structure and dynamics [28].

To generate a conformational ensemble of chromosomes, we used the low friction Langevin simulation [46] (see S1 Text) and sampled the folded conformations of chromosome by collapsing an ensemble of extended chromatin chains. The conformational ensemble of Chr10, resulting from the enhanced sampling of chromosome conformation, produces a checkerboard pattern which resembles that of the Hi-C contact map [9] (Fig 1A), and it displays the hallmark of space-filling (SF) statistics, i.e., the characteristic scaling of contact probability *P*(*s*) ∼ *s*^−1^ over the intermediate range of genomic distance 1 < *s* < 10 Mb (S1B Fig). The distribution of Alexander polynomial, |Δ(*t* = −1)| [47](S1D Fig), which characterizes the amount of chain entanglement, has the highest mode at |Δ(*t* = −1)| ≈ 0, which indicates that the majority of chromosome conformations are free of knots. According to the radial distributions of monomers belonging to the different subcompartment types [22, 48], the condensed and transcriptionally inactive loci are buried inside the chromosome, whereas the open and active loci are distributed near the chromosome surface, which is presumably needed to increase the accessibility to transcription factors (S1E and S1F Fig).

Because of the nature of frustrated interactions in the heteropolymer model, substantial heterogeneity is expected for the structural ensemble; thus rigorous conformational sampling is not easy to achieve. Nonetheless, the resulting heterogeneity of conformational ensemble can be visualized using clustering analysis over the structures generated. In order to quantify the (dis)similarity between two conformations and to perform the clustering analysis for the structures, we use the distance-based root-mean-square deviation (DRMS, D),

$$\begin{array}{c} D_{\alpha ,\beta }=\sqrt{\frac{2}{N(N-1)}\sum_{i>j}{\left(r_{i,j}^{\alpha }-r_{i,j}^{\beta }\right)}^{2}}. \end{array}$$ (1)

If DRMS of two distinct chromosome structures, say *α* and *β*, is smaller than a cut-off value $D_{c}$ such that $D_{\alpha ,\beta }<D_{c}$, we consider them similar and group them together into the same cluster. By repeating this grouping process with increasing value of $D_{c}$ we clustered the chromosome structures hierarchically; the result is summarized into a dendrogram (Fig 1B and S2 Fig). When $D_{c}$ reaches 〈D〉≈4.5a, which corresponds to the average DRMS, the distinction between the structures belonging to different clusters or between their contact maps becomes clear (Fig 1B). We will show that the transformation of a conformation in one cluster to those in another cluster beyond the value of DRMS greater than 〈D〉 is dynamically a very slow process. Partitioning of the conformations into distinct clusters is a first indication that the configurational space of chromosome is rugged, suggestive of the cell-to-cell variability discovered in the recent single-cell Hi-C data [5, 49, 50].

### Dynamics of chromatin loci probed with mean square displacement

The time-averaged mean square displacement (MSD) is a routinely calculated quantity in analyzing the dynamics of cellular constituents in live cell imaging experiments as well as in chromosome studies [44, 51, 52].

**Fig 2:**
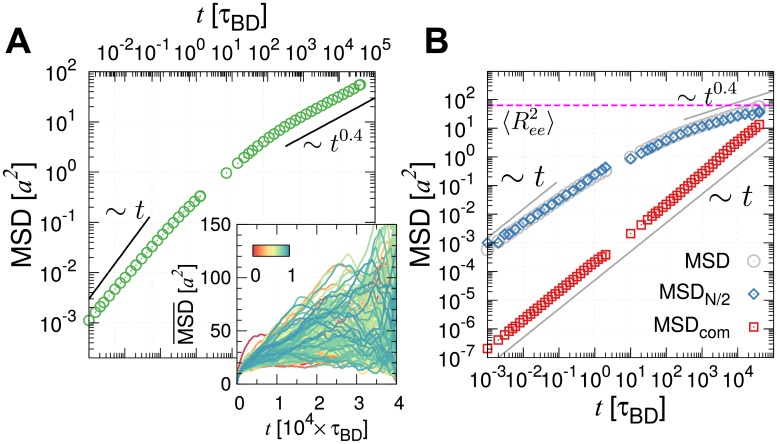
Subdiffusive behavior of chromatin loci. (A) Loci- and time-averaged MSD generated from a single time trajectory in a log-log plot (Inset displays the time-averaged MSD for individual loci, color-coded by a normalized monomer index *i*/*N*.). (B) Time-averaged MSD of the midpoint monomer ${\bar{\text{MSD}}}_{N/2}$, and the center of mass of the whole chain ${\bar{\text{MSD}}}_{\text{com}}$ in a log-log plot. The horizontal dashed line labels the mean square end-to-end distance $\langle R_{ee}^{2}\rangle$ (= 62.7 ± 0.9 *a*^2^) of the chromatin chain. Conversion to the physical time can be made using *τ*_BD_ ≈ 50 ms.

The time-averaged MSD for *i*-th locus is defined as ${\bar{\text{MSD}}}_{i}\left(t\right)=\langle |{\vec{r}}_{i}\left(t_{0}+t\right)-{\vec{r}}_{i}\left(t_{0}\right){|}^{2}\rangle_{t_{0}}=\frac{1}{\tau_{\text{max}}-t}\int_{0}^{\tau_{\text{max}}-t}dt_{0}{\left|{\vec{r}}_{i}\left(t_{0}+t\right)-{\vec{r}}_{i}\left(t_{0}\right)\right|}^{2}$, where *τ*_max_ (= 4 × 10^4^*τ*_BD_ ∼ 0.5 hour: see Methods) is the longest simulation time. The loci-averaged MSD is then obtained by summing over the loci as $\text{MSD}\left(t\right)=\sum_{i=1}^{N}{\bar{\text{MSD}}}_{i}\left(t\right)/N$. Substantial dynamical heterogenetiy is present in ${\bar{\text{MSD}}}_{i}\left(t\right)$ for different *i* (the inset of Fig 2A and S3 Fig). As a result, the dynamics of individual loci is characterized with a different scaling exponent *β* at long time (see S3 Fig). Dynamics of individual locus, quantified in terms of ${\bar{\text{MSD}}}_{i}\left(t\right)$ depends on the position of locus and varies from one trajectory to another. Nevertheless, the diffusion of chromatin loci is on average characterized by three different time regimes (Fig 2A). (i) At short times (*t* < 10^−2^*τ*_BD_), the loci diffuse freely with MSD ∼ *t*. (ii) At the intermediate times, corresponding to the Brownian time *t* ∼ *τ*_BD_ ∼ *a*^2^/*D*, each locus starts to feel the influence of adjacent loci. (iii) For *t* > 10^3^ *τ*_BD_, a subdiffusive behavior of MSD ∼ *t*^*β*^ with *β* ≈ 0.4, spanning at least 2–3 orders of time interval, is observed (Fig 2A). This exponent is in line with the reported values of *β* = 0.38 ∼ 0.44 [45] and *β* = 0.4 ∼ 0.7 [13] from live human cells.

As discussed in other studies [45, 53], the exponent *β* = 0.4 of loci-averaged MSD at *t* > 10^3^*τ*_BD_ can be rationalized using the following argument. The spatial distance (*R*) between two loci separated by the curvilinear distance, *s*, satisfies *R*(*s*) ∼ *s*^*ν*^, where *ν*, the scaling exponent [42, 54], is *ν* = 1/2 for the ideal chain obeying the random walk statistics, and *ν* = 1/3 for the space-filling (SF) chain for crumpled globules. Notice that the MSD of a locus in a chain segment of arc length *s* scales with time *t* as MSD ∼ *t*^*β*^ ∼ *D*(*s*) × *t* ∼ *D*_*o*_ × *t*/*s*, where the scaling relationship of the diffusion constant of freely draining chain *D*(*s*) ∼ *D*_*o*_/*s* is used. Meanwhile, the space taken up by the chain segment of arc length *s* is described by the relation of MSD ∼ *R*^2^(*s*) ∼ *s*^2*ν*^. These two relations of MSD allow us to relate *s* with *t* as *s* ∼ *t*^*β*/2*ν*^, and it follows that MSD ∼ *t*^*β*^ ∼ *t*^1−*β*/2*ν*^, which leads to *β* = 2*ν*/(2*ν* + 1) [45, 53]. Thus we obtain

$$\begin{array}{c} \text{MSD}\left(t\right)∼t^{\frac{2\nu }{2\nu +1}}. \end{array}$$ (2)

The SF organization of chromosome at intermediate scales (1 ≪ *s* < *N*^2/3^) implies *ν* = 1/3, and hence *β* = 0.4. A similar argument was used to explain the growth of MSD(*t*) in an entirely different model [39]. Other theories [45, 55] and a modeling study [26], which consider interactions to maintain the compactness of the chain structure, lead to the same conclusion.

Meanwhile, a high-throughput measurement of chromatin motion tracking has shown MSD ∼ *t*^0.5^ for yeast chromosomes [11]. Evidently, MSD ∼ *t*^1/2^ for *ν* = 1/2 from Eq 2, and it is well known that yeast chromosomes obey the random walk statistics (*R*(*s*) ∼ *s*^1/2^ and *P*(*s*) ∼ *s*^−3/2^), indicative of *ν* = 1/2. Therefore, the diffusion exponent of chromosome loci reflects the effect of chain organization of chromatin in chromosome structure [45, 53, 55].

The loci-averaged MSD(*t*) is used as a handy probe for chromatin dynamics in experiments [45, 53, 55]. However, when a polymer is *extraordinarily* long just like in the problem of chromatin chain, ${\bar{\text{MSD}}}_{i}\left(t\right)$ of the *i*-th locus of even a *homopolymer* depends critically on the position of the locus and its motion exhibit its characteristic scaling behavior at different time regimes with various crossovers [26, 27, 56–58]. The scaling behavior of ${\bar{\text{MSD}}}_{i}\left(t\right)$ for different loci (different *i*) at different time regimes can be used to disentangle the dynamics of a polymer chain, e.g., the diffusion time along the tube that can be hypothesized in melt-like dense polymer environment ($\tau_{e}=N_{e}^{2}/W$), Rouse relaxation time (*τ*_*R*_ = *N*^2^/*W*), and reptation time (*τ*_*N*_ = *N*^3^/*N*_*e*_*W*), where *N*_*e*_ and *W* denotes the entanglement length and diffusivities of polymer segments, respectively. A test polymer chain of length *N* in a highly entangled equilibrium melt (*N*_*e*_ < *N*) [56–58], exhibits scale-dependent dynamics with multiple crossovers:

$$\begin{array}{c} {\bar{\text{MSD}}}_{N/2}∼t^{1/2}\text{,}\;{\bar{\text{MSD}}}_{\text{com}}∼t\;\text{for}\;t<\tau_{e};\\ {\bar{\text{MSD}}}_{N/2}∼t^{1/4}\text{,}\;{\bar{\text{MSD}}}_{\text{com}}∼t^{1/2}\;\text{for}\;\tau_{e}<t<\tau_{R};\\ {\bar{\text{MSD}}}_{N/2}∼t^{1/2}\text{,}\;{\bar{\text{MSD}}}_{\text{com}}∼t\;\text{for}\;\tau_{R}<t<\tau_{N};\\ {\bar{\text{MSD}}}_{N/2}∼t\text{,}\;{\bar{\text{MSD}}}_{\text{com}}∼t\;\text{for}\;\tau_{N}<t. \end{array}$$ (3)

where the behaviors of time-averaged MSDs were given for the mid-point monomer (*i* = *N*/2) and the center of mass (*i* = com). Our chromosome model differs from polymer melts and thus the above scalings of ${\bar{\text{MSD}}}_{i}\left(t\right)$ for an ideal test chain (*ν* = 1/2) in polymer melts in principle do not apply to our chromosome model comprised of *non-ideal* subchains (*ν* = 1/3). Nevertheless, the crossover behaviors at distinct characteristic times (*τ*_*e*_, *τ*_*R*_, *τ*_*N*_) discussed in Eq 3 is still be of great use to illuminate the dynamics of our chromosome model.

Two points are worth making. (i) The distribution of Alexander polynomial indicates that our chromatin chain is rarely entangled (S1D Fig). Thus *τ*_*e*_ is not a quantity relevant to our chromosome model. Furthermore, ${\bar{\text{MSD}}}_{\text{com}}∼t$ for the entire simulation time (Fig 2C), which is also an indication of the absence of the crossover. (ii) For an ideal Rouse chain, the chain relaxation time (the Rouse time, *τ*_*R*_) can be estimated from ${\bar{\text{MSD}}}_{\text{com}}=\langle R_{ee}^{2}\rangle$ at *t* = 3*τ*_*R*_/4, where $\langle R_{ee}^{2}\rangle$ is the mean square end-to-end distance of the chain [57, 58]. In our case, MSD of ‘com’ still has not reached $\langle R_{ee}^{2}\rangle$ even at the maximum simulation time, i.e., ${\bar{\text{MSD}}}_{\text{com}}\left(t=\tau_{\text{max}}\right)<\langle R_{ee}^{2}\rangle$, which indicates that the total simulation time of our study is still shorter than the Rouse relaxation time (*τ*_max_ < *τ*_*R*_).

Taken together, the two critical time scales for equilibration, the reptation and Rouse relaxation times, of our model are substantially longer than the typical time scales relevant for cellular processes such as cell doubling times (see below). The global dynamics of chromosomes are not only heterogeneous but also are too slow for a full equilibration. Thus, it is reasonable to view that chromosome dynamics is sluggish, glass-like and occurs out of equilibrium.

### Correlated loci motion in space and time

Correlation functions are a general tool to study the dynamics of complex systems [59], and have been used in experimental analysis of genomes or chromosomes [10, 12, 60, 61]. Here, we adopt this strategy to study the spatio-temporal dynamics of our chromosome model.

**Fig 3:**
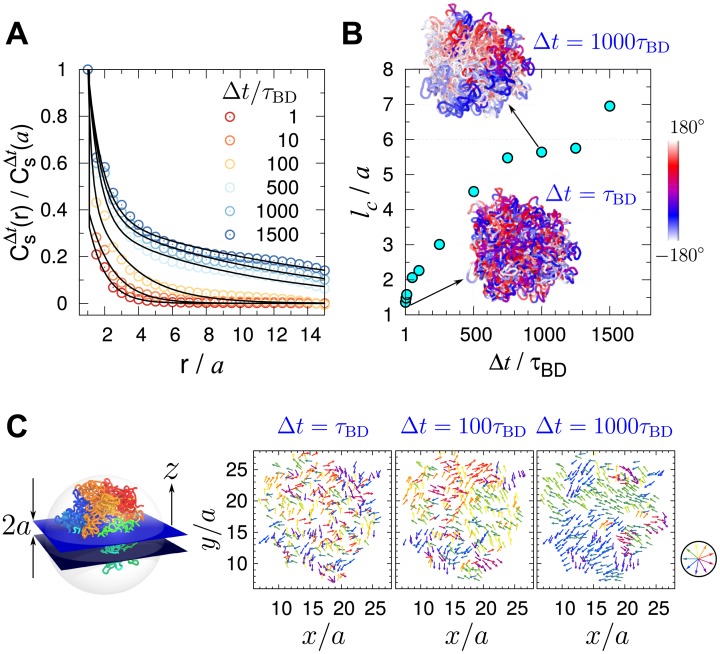
Spatial correlation between loci displacements. (A) Spatial correlation of loci displacements $C_{s}^{\Delta t}\left(r\right)$ (Eq 4) with varying lag time (Δ*t*). (B) Correlation length $l_{c}(=\int_{0}^{\infty }\left[C_{s}^{\Delta t}\left(r\right)/C_{s}^{\Delta t}\left(a\right)\right]dr)$ as a function of Δ*t*. Visualized on 3D chromosome structure are the displacement correlations of chromatin loci probed at short and large time gap (Δ*t* = *τ*_BD_ and 10^3^ *τ*_BD_) projected onto the xy-plane. The color-code on the structures depicts the azimuthal angle of loci displacement. (C) The displacement vector of loci in the equator plane are color-coded by direction. In each panel, the displacement vectors $\Delta \vec{r}\left(t=0;\Delta t\right)$ are calculated for Δ*t* = 1, 100, 1000 *τ*_BD_. Direction-dependent color scheme is depicted on the right.

Recently, displacement correlation spectroscopy (DCS) using fluorescence has been employed to study the dynamics of whole chromosomes in the nucleus, revealing that coherent motion of the *μ*m-sized chromosome territories could persist for *μ*s to tens of seconds [10]. We adopted the same approach used in DCS and studied the spatial correlation in the intra-chromosomal dynamics generated from our simulations. The spatial correlation between chromatin loci is evaluated using

$$\begin{array}{c} C_{s}^{\Delta t}\left(r\right)={\langle \frac{\sum_{i>j}\left[\Delta {\vec{r}}_{i}\left(t;\Delta t\right)\cdot \Delta {\vec{r}}_{j}\left(t;\Delta t\right)\right]\delta \left(r_{i,j}\left(t\right)-r\right)}{\sum_{i>j}\delta \left(r_{i,j}\left(t\right)-r\right)}\rangle }_{t}, \end{array}$$ (4)

which quantifies the displacement correlations between loci separated by the distance *r* over the time interval Δ*t*. $C_{s}^{\Delta t}\left(r\right)$ decays more slowly with increasing Δ*t*. The correlation length calculated using $l_{c}=\int_{0}^{\infty }\left[C_{s}^{\Delta t}\left(r\right)/C_{s}^{\Delta t}\left(a\right)\right]dr$, shows how *l*_*c*_ increases with Δ*t* (Fig 3B). To demonstrate an image of displacement correlation over the structure, we project the displacement vectors of the monomers near the equator of the confining sphere (−*a* ≤ *z* ≤ *a*) onto the *xy* plane, and visualize the dynamically correlated loci moving parallel to each other by using the vector field with a similar color (see Fig 3C). If Δ*t* < 100 *τ*_BD_, the spatial correlation of loci dynamics is short-ranged and the displacement vectors appear to be random. In contrast, multiple groups of coherently moving loci that form substantially large domains (∼ 5*a* ≈ 0.75 *μm*) emerge at a longer waiting time (Δ*t* > 500 *τ*_BD_).

We also calculated $C_{s}^{\Delta t}\left(r\right)$ for the Rouse chain as a reference (see SI). Just like our chromosome model, $C_{s}^{\Delta t}\left(r\right)$ for the Rouse chain decays more slowly over the distance *r* with increasing Δ*t* (S4A Fig), and the correlation length *l*_*c*_ increases *monotonically* with Δ*t* as well (S4B Fig). However, this very feature differs from the one observed in the experiment [10] where *l*_*c*_ displayed *nonmonotonic* change with Δ*t*. In fact, the experimentally observed nonmonotonic change of *l*_*c*_ is obtained by incorporating active noise to the model, which will be discussed in the section that follows (see below, **Effects of active noise on chromosome dynamics**).

In parallel to the spatial correlation functions calculated above, a time-correlation function that can potentially characterize the chromatin dynamics has recently been proposed [12, 60] for the displacement vectors of the same locus or two distinct loci for varying lag times. However, we find the resulting time-correlation function (mean velocity auto-correlation function) is not so informative in the sense that it is barely discernible from that of the ideal Rouse chain (see S1 Text and S5 Fig for details).

### Euchromatin versus heterochromatin dynamics

**Fig 4:**
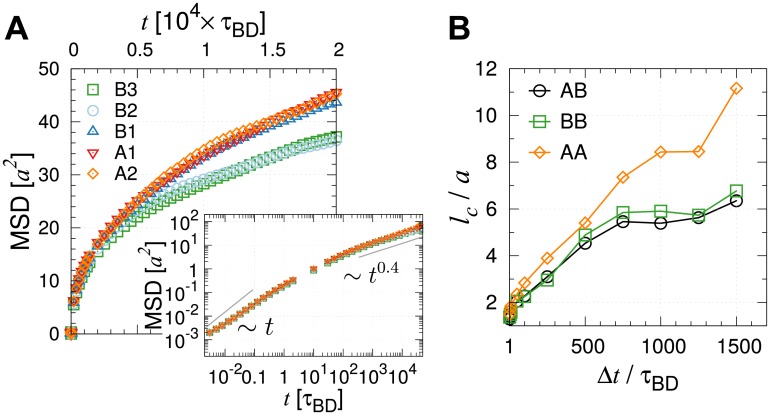
Dynamics of different sub-compartment types of loci. (A) Shown are the loci-averaged MSDs of A and B type loci. The log-log plot in the inset indicates that the diffusion exponent *β* is effectively identical for the A and B type loci. The locus-dependent dynamics is provided in S3 Fig. (B) Correlation length as a function of Δ*t* calculated from spatial correlation functions $C_{s,AB}^{\Delta t}\left(r\right)$, $C_{s,BB}^{\Delta t}\left(r\right)$, $C_{s,AA}^{\Delta t}\left(r\right)$ between different types of loci AB, BB, and AA, respectively.

Diffusion of heterochromatin-rich loci is slower than euchromatin-rich loci [45]. The time-averaged MSD (${\bar{\text{MSD}}}_{i}$) exhibits substantial dispersion among different loci (Fig 2A inset and S3 Fig), and the overall mobility of loci depends on the subcompartment types (see Fig 4A). In our chromosome model we find that the A-type loci, which are less condensed and distributed closer to the chromosome surfaces, diffuse faster than the B2 and B3 type loci. The dispersion of ${\bar{\text{MSD}}}_{i}$ shown in the inset of Fig 2A is the outcome of both different sub-compartment types and different genomic positions of loci. Although the diffusivity is greater for the active loci, they still have the same *β* = 0.4 for *t* > 10^3^*τ*_BD_ (Fig 4A inset). The relation *β* = *β*(*ν*) = 2*ν*/(2*ν* + 1) suggests that the exponent *ν* representing the chain organization is the sole determinant of the diffusion exponent (*β*) characterizing the global motion. We will show that this conclusion holds good even in the presence of active noise incorporated into the chromatin dynamics (see below).

Decomposing the spatial correlation $C_{s}^{\Delta t}\left(r\right)$ into A and A, B and B, or A and B type loci (S6A Fig), we find that the corresponding correlation length *l*_*c*_ of A-type loci is greater than B-type loci for Δ*t* ≳ *τ*_BD_ (Fig 4B). This suggests that the motion of A-type loci is more coherent; however, this picture changes completely when “activity” is incorporated into the model (see below).

### Relaxation times of chromatin dynamics depend on the length scale

**Fig 5:**
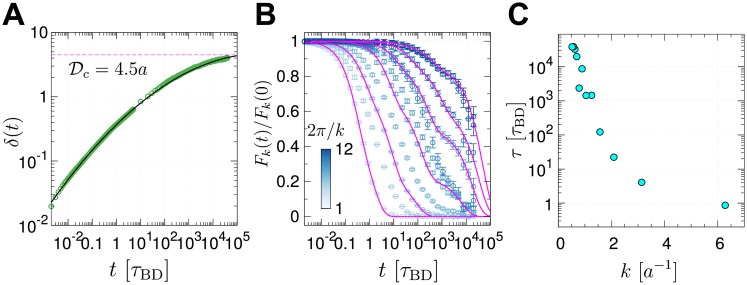
Relaxation times of chain conformations. (A) Time evolution of the root mean square distance between a pair of loci *r*_*i*,*j*_(*t*) at time *t* relative to its initial value (*r*_*i*,*j*_(0)) averaged over all pairs, defined by *δ*(*t*) = [2∑_*i*>*j*_(*r*_*i*,*j*_(*t*) − *r*_*i*,*j*_(0))^2^/*N*(*N* − 1)]^1/2^. (B) Normalized intermediate scattering function *F*_*k*_(*t*)/*F*_*k*_(0), with different values of wave number *k*, were calculated from BD simulation trajectories of the chromosome. (C) The chain relaxation time (*τ*) for different wave number *k* was estimated by evaluating $\tau_{k}=\int_{0}^{\infty }\left[F_{k}\left(t\right)/F_{k}\left(0\right)\right]dt$.

The time evolution of the averaged mean square deviation of the distances between two loci with respect to the initial value (see Fig 5A and the caption for the definition of *δ*(*t*)) was calculated to discuss the dynamical stability of chromosome structure. Within our simulation time *τ*_max_, the largest value *δ*_max_(= 4.0 ± 0.3 *a*) is smaller than the value, $D_{c}=4.5$ *a*, which was chosen to define different conformational clusters in Fig 1B. An extrapolation of *δ*(*t*) to $\delta \left(\tau_{c}\right)=D_{c}$ gives an estimate of *τ*_*c*_ ≈ 10^5^ × *τ*_BD_ ≈ 1.4 hours, which is a long time scale considering that most cells of adult mammals spend about 20 hours in the interphase [62].

From the definition of, *δ*(*t*), it follows that lim_*t*→∞_〈*δ*(*t*)〉 = *δ*_eq_. Here, *δ*_eq_ is finite, and 〈⋯〉 is an ensemble average, meaningful only if the equilibrium is reached. We estimate *δ*_eq_ assuming that the long time limit of the mean deviation of the distance between two loci is approximately the mean end-to-end distance between the loci. Thus, $\lim_{t\to \infty }\langle {\left(r_{ij}\left(t\right)-r_{ij}\left(0\right)\right)}^{2}\rangle ∼R_{ij}^{2}$ where *R*_*ij*_ is the mean end-to-end distance between *i*^*th*^ and *j*^*th*^ loci. For |*i* − *j*| ≫ 1, we expect that $R_{ij}^{2}∼a^{2}{\left|i-j\right|}^{2\nu }$. Consequently, *δ*_eq_ can be calculated using $\delta_{\text{eq}}^{2}=\frac{2}{N(N-1)}\sum_{s=1}^{N-1}\left(N-s\right)R^{2}\left(s\right)=\frac{2a^{2}}{N(N-1)}\sum_{s=1}^{N-1}\left(N-s\right)s^{2\nu }$. For *N* = 2712, and with *ν* = 1/3 we estimate *δ*_eq_ ≈ 9.4 *a*, which is greater than the value (*δ*_max_ ≈ 4.0 *a*) reached at the longest times (Fig 5A). An upper bound of *δ*_eq_ for an ideal Rouse chain is 16.4 *a* (see SI). These considerations suggest that the chromosome dynamics falls short of equilibrium on the time scale of a single cell cycle.

Relaxation dynamics of chromatin domain should be scale-dependent, which is quantified using the time evolution of intermediate scattering function *F*_*k*_(*t*) [59, 63], the van Hove correlation function in Fourier space, calculated at different length scale (∼ 2*π*/*k*) (Fig 5B):

$$F_{k}\left(t\right)={\langle {\langle \frac{1}{N}\underset{m}{\sum }e^{i\vec{k}\cdot {\vec{r}}_{m}\left(t+t_{0}\right)}\underset{n}{\sum }e^{-i\vec{k}\cdot {\vec{r}}_{n}\left(t_{0}\right)}\rangle }_{\left|\vec{k}\right|}\rangle }_{t_{0}},$$ (5)

where ${\langle {\langle \ldots \rangle }_{|\vec{k}|}\rangle }_{t_{0}}$ is an average over *t*_0_ and over the direction of vectors $\vec{k}$ with magnitude $k(=|\vec{k}|)$. Two points are worth making for *F*_*k*_(*t*) at varying *k*. (i) The chromatin chains at high wave number (at local scale) relax fast, which implies that chromatin chains are locally fluid-like (2*π*/*k* ≲ *a*). Although the structure of TAD is highly coarse-grained in our study (TADs, whose median size is 880 Kb [7], is represented by only 18 beads), this fluid-like dynamics at local scale is in accord with the recent experimental finding on the structural deformation of chromatin fibers within TADs [8, 64]. (ii) The spatial organizations of chromatin chains over intermediate to global scales (2*π*/*k* ≫ *a*) are characterized by slow relaxation dynamics. This scale-dependent relaxation time is reminiscent of a similar finding in random heteropolymers [65].

**Fig 6:**
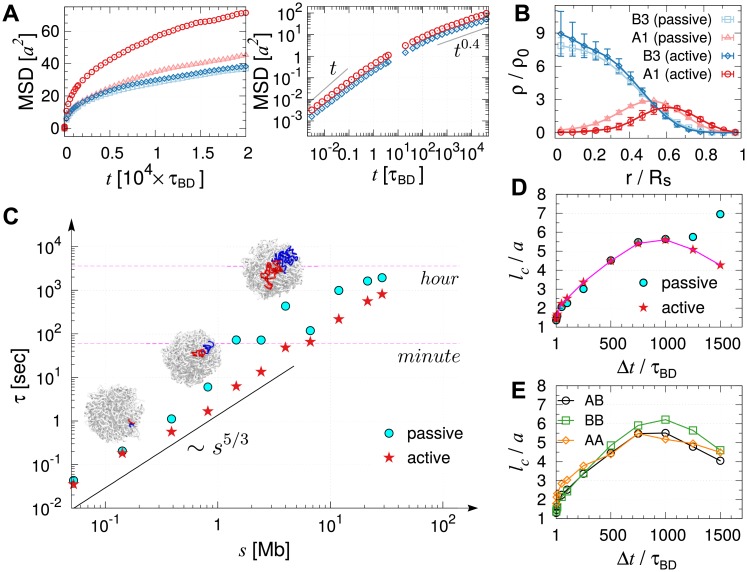
Effects of active noise on chromosome organization and dynamics. (A) MSD of active and inactive loci compared with those under passive condition. Log-log plot is shown in the panel on the right. (A) shares the same legend with (B). (B) Distribution of active (A1) and inactive (B3) loci with and without active noise. In the presence of active noise, the segregation of active and inactive loci is more evident. (C) Relaxation times estimated from the intermediate scattering functions. The wave number *k* was mapped to the corresponding number of loci inside the volume defined by the wave number. The red star symbols, the relaxation times in the presence of active noise, are depicted for the comparison with those under passive condition. (D) Correlation lengths for varying Δ*t* calculated using the loci displacement correlations under passive (Fig 3A) and active (S7 Fig) conditions are compared. (E) Correlation length calculated for different types of loci from spatial correlation functions, $C_{s,AB}^{\Delta t}\left(r\right)$, $C_{s,BB}^{\Delta t}\left(r\right)$, $C_{s,AA}^{\Delta t}\left(r\right)$, in the presence of active noises.

Relaxation time (*τ*) of a subdomain of size *ξ* = 2*π*/*k* is estimated using $\tau_{k}=\int_{0}^{\infty }\left[F_{k}\left(t\right)/F_{k}\left(0\right)\right]dt$, which can in turn be related to the number of coarse-grained monomers comprising the subdomain as *ξ* ∼ 2*π*/*k* ∼ *s*^*ν*^. Since the chromosome domain loses memory of the initial conformation by spatial diffusion (instead of reptation), the relaxation time *τ* is expected to obey *τ* ∼ *ξ*^2^/*D*_eff_ ∼ (*s*^*ν*^)^2^/(*D*_0_/*s*) ∼ *s*^2*ν*+1^, thus *τ* ∼ *s*^5/3^ for the chromosome structure that obeys SF statistics (*ν* = 1/3). The size-dependent relaxation times calculated for our chromosome model indeed scales with the domain size as *τ* ∼ *s*^5/3^ (cyan symbols and solid line in Fig 6C).

### Effects of active noise on chromosome dynamics

Effects of biological activities on the chromosome structure, such as ATP hydrolysis-driven non-conservative forces exerted by cohesins [20], are only implicit in the original MiChroM in terms of the differential energy parameters for the loci of A, B subcompartment types. Thus, it could still be argued that such a model misses the most critical component of living systems. Live cells abound in a plethora of biological activities such as replication, transcription, and error-correcting dynamics. While these processes produce local directionality, when mapped onto our model that has coarse-grained 50 Kb of DNA into a single bead, the effects of vectorial forces on the surrounding environment at length and time scales greater than the correlation length and time of active noises can be assumed *isotropic*. This is supported by Javer *et. al*. [66] who also pointed out, by performing an experimental study of locus-dependent diffusion coefficient in *E. coli*., that the contribution of “ballistic” motion to MSD beyond the time scale of *seconds* is negligible. We study how an increased noise strength on the active loci (A1 and A2) occupying 40% of loci population for Chr10, which resuts in the breakdown of fluctuation-dissipation theorem [67, 68], affects the dynamical properties of entire chromosome. To model the active noise, we increased the noise strength from $\langle {\vec{R}}_{i}\left(t\right)\cdot {\vec{R}}_{j}\left(t^{\prime }\right)\rangle =6D_{i0}\delta_{ij}\delta \left(t-t^{\prime }\right)$ to $\langle {\vec{R}}_{i}\left(t\right)\cdot {\vec{R}}_{j}\left(t^{\prime }\right)\rangle =12D_{i0}\delta_{ij}\delta \left(t-t^{\prime }\right)$, following the recent literature [69, 70].

The model that incorporates active noises as described above has led to two important results. (i) The disproportionate increase in the mobility of A and B type loci promotes the phase segregation of the two loci types (see Fig 6B, S3 Fig, and compare S1 and S2 Movies). The active noises push A-type loci towards the surface of the chromosome, and B-type loci are pulled towards the center to offset this effect. (ii) More quantitatively, we find that the average MSD of A1 loci exhibits ∼ 70% increase relative to the passive case (Fig 6A left panel), while the diffusion exponent (*β* ≈ 0.4 in MSD ∼ *t*^*β*^) remains unaltered (Fig 6A right panel and S3 Fig). The finding that the inclusion of active noises increases the amplitude of the MSD without altering the diffusion exponent (*β* ≈ 0.4) is in accord with an experiment on bacterial chromosomes performed with and without ATP depletion [44]. In addition, the finding is consistent with the MSD data reported for a live human Hela cell [45], where chromatin loci at the nuclear periphery and interior, corresponding to the heterochromatin and euchromatin, displayed diffusion exponents *β* = 0.39 and 0.41, respectively, although the MSD of the euchromatin was significantly greater. We however also note that the diffusion exponent *β* = 0.32 ± 0.03 was reported for the whole genome of ATP-depleted HeLa cells [10], which is qualitatively different from *β* ≈ 0.4 (see S1 Text and S8 Fig for detailed analyses of the experimental data reported in [10]).

In terms of *F*_*k*_(*t*), the active noises mainly influence the chain relaxation associated with the low frequency modes. For the high frequency modes or at local length scales (*k* ≳ 2*π*/3*a*), *F*_*k*_(*t*) is practically indistinguishable between active and passive cases (S9 Fig). The chromatin segments in the presence of active noise, on average, relax faster when the size of the segment is greater than the sub-Mb. A comparison of the relaxation times in Fig 6C under passive and active conditions highlight this difference.

Similarly, the effect of active noise on the correlation length (*l*_*c*_) is evident only at a large lag time (Δ*t*). We find that in contrast to the passive case, *l*_*c*_ changes nonmonotonically with Δ*t*. There is no distinction between the effects of passive and active noises on *l*_*c*_ for small Δ*t*; however, deviation between the two cases becomes evident for Δ*t* ≳ 10^3^*τ*_BD_ ≈ 50 sec (Fig 6D). Importantly, a similar dependence of correlation length on Δ*t* has been discussed in DCS measurement on genome-wide dynamics of live cell [10].

To dissect the contribution from the loci of each subcompartment type in the presence of active noises, we again calculated the spatial correlation $C_{s,AB}^{\Delta t}$, $C_{s,BB}^{\Delta t}$, $C_{s,AA}^{\Delta t}$ (S6B Fig) and the corresponding correlation lengths (*l*_*c*_) (Fig 6E). At short time scale (*t* < 500*τ*_BD_), A-type loci display slightly stronger self-correlations than B-type loci. In stark contrast to the passive case (Fig 4B), however, at Δ*t* > 500*τ* active noises disturb the spatial correlations between active loci, which subsequently reduces the correlation of entire structure. Compared to the thermal noise (Fig 4B), the active noises randomize the global structure of chromatin chain more efficiently, which shortens the correlation length at sufficiently large lag time.

## Discussion

Despite a great amount of complexity inherent to its size and heterogeneous interactions that give rise to various dynamic behaviors at different time and length scale and crossovers, chromatin chain folded into a heterogeneous ensemble of chromosome conformations via protein mediated interactions can be viewed from a perspective of polymer physics as a *very long* heteropolymer chain collapsed in a poor solvent condition [56–58]. Our study highlights the importance of chromosome architecture in determining the subdiffusive behavior and dynamic correlations between distinct loci. Most importantly, we have shown that *structure* alone explains many of the dynamical features observed in live cell experiments [10, 13, 44, 45]. In other words, conformational properties of chromatin chain dictate the dynamics of chromosome. Remarkably, several static and dynamic properties of the model, including *R*(*s*) ∼ *s*^*ν*^, *P*(*s*) ∼ *s*^−3*ν*^, MSD(*t*) ∼ *t*^2*ν*/(2*ν*+1)^, *τ*(*s*) ∼ *s*^2*ν*+1^, and $\langle X_{p}^{2}\rangle ∼p^{-(1+2\nu )}$ (${\vec{X}}_{p}$ is the *p*-th Rouse mode. See S1 Text and S5D Fig for the details) are fully explained by the SF organization characterized by the single scaling exponent *ν* = 1/3, offering a unified perspective on the link between the structure and dynamics of chromosomes.

The relaxation time (*τ*) of the chromatin domain spans several orders of magnitude depending on its genomic length (*s*), satisfying the scaling relation *τ* ∼ *s*^5/3^ (Fig 6C). To be more concrete (see Fig 6C), while local chromatin domains of size s≲O(1) Mb, a scale corresponding to TADs, reorganize on the time scale of $t<10^{3}\tau_{\text{BD}}∼O\left(1\right)$ seconds, it takes more than hours to a day for an entire chromosome chain (≳ 100 Mb) to lose its memory of the initial conformation as long as the chromosomes are in the interphase with no significant vectorial active noises. This timescale of relaxation is expected to increase even further at higher volume fractions [28]. Under *in vivo* conditions, with 46 chromosomes segregated into chromosome territories, the time scale for relaxation would be considerable.

The effects of active noise on chromatin dynamics [10, 44] deserve further discussion. While the isotropic active noises modeled in this study enhance chain fluctuations and structural reorganization, their effect on chromatin domain manifests itself only on length scales greater than 5.5 *a* (≈ 0.8 *μm*), and on a time scale greater than 50 sec (Fig 6D). Our finding is reminescent of the microrheology measurements on active cytoskeletal network [71], where the effect of myosin activity could be observed only at low frequency regime of the loss modulus. Of course, the active noise in live cell nuclei is still not a scalar, and thus it remains a challenge to model their vectorial nature in the form of force dipole or vector force in the context of chromatin dynamics [69]. Vector activities promote super-diffusive motion (${\bar{\text{MSD}}}_{i}∼t^{\beta }$ with 1 < *β* < 2), and could in principle elicit a qualitative change in the dynamical scaling relations as in the mitotic phase. Still, the dynamic scalings discussed in this study (e.g., MSD∼ *t*^0.4^) are in good agreement with those observed in interphase chromatins of live cells [13, 45]. There could be many different explanation for this observation, but we reason as follows. In terms of *power* generated in a cell, the passive (thermal) power *W*_*p*_ ∼ *k*_*B*_*T*/ps is many orders of magnitude greater than the active power (e.g., molecular motors, *W*_*a*_ ∼ 20 *k*_*B*_*T*/10 ms [62]). At least in the *interphase*, the gap between the total passive and active power is substantial; the number of active loci (*N*_*a*_) is smaller than the number of passive loci (*N*_*p*_), rendering the total passive power much greater than the active power (*N*_*p*_*W*_*p*_ ≫ *N*_*a*_*W*_*a*_). Thus, the total energetic contribution of the biological activities during the interphase to the chromosome structure would be insignificant compared to thermal agitation. Taken together, even in the presence of biological activities, as long as the scaling exponent *ν* = 1/3 characterizing the chromosome structure is unaltered, the various dynamical scaling behaviors remain intact.

To recapitulate, we have shown that the SF organization (*ν* = 1/3) adopted by a block-copolymer type model of chromosome alone suffices to explain many of the experimentally observed loci dynamics of human interphase chromosomes. The average behaviors of chromatin dynamics that we have drawn here should not depend critically on the details of the chromosome model being used. One should be able to confirm them as long as a chromosome designed using those models maintains crumpled architectures displaying SF statistics with *ν* = 1/3. On one hand, despite seemingly a daunting problem at first sight, many aspects of chromosome dynamics can be quantitatively explained and predicted using purely physical argument based on the basic concepts of polymer physics. This means that if care is taken, even the dynamics of a highly complex biological object like chromosome can be deciphered using the physical law as far as the global dynamics averaged over the large ensemble is concerned. On the other hand, experimental measurement should either be made at a higher resolution in space and time or be specific to genomic loci in individual cells, if one were to extract dynamical information relevant for specific biological function of chromosomes beyond the fractal dimension of chain organization.

## Methods

To build the model of chromosome 10 of human lymphoblastoid cell and study its dynamical behaviors, we used the energy potentials and parameters of MiChroM, a type of block-copolymer (heteropolymer) model. The coarse graining of chromatin leads to *N* = 2712 loci with the diameter of each locus being *a* ≈ 150 nm, so that a single locus represents 50 Kb of DNA. The inverse mapping of the Hi-C map to the ensemble of chromosome structures was carried out by sampling the conformational space using low-friction Langevin simulations [46]. The generated structures exhibit the characteristic scaling of the contact probability, *P*(*s*) ∼ *s*^−1^, and show the spatial distribution of A/B compartment as well as the plaid pattern noted in Hi-C experiments. Whereas the original study of MiChroM allowed the chain-crossing with an energetic penalty for the purpose of sampling the conformations whose population reproduces the Hi-C map, we imposed a strict chain non-crossing constraint on the chromosome structures and performed Brownian dynamics simulations to study the dynamics of chromatin when the conformational sampling was completed.

The mapping from simulation times to the physical times deserves a few remarks. The apparent viscosity of nuclear environment varies among different experimental reports within an order of magnitude: *η* = 1–3 cP [72], 3 cP [73], 7 cP [74], and 10 cP was assumed in modeling chromosome dynamics [32]. In the model employed in this study, each monomer represents 50 Kb genomic region, which is mapped to the diameter of *a* = 150 nm. Assuming that the nuclear viscosity *η* = 7 cP, the Brownian time of single particle *τ*_BD_ = 3*πηa*^3^/*k*_*B*_*T* ≈ 50 ms. Therefore, the longest simulation time in this study *τ*_max_ = 4 × 10^4^ *τ*_BD_ corresponds to 0.5 hour. At 0.5 second, MSD measured in the nucleus of HeLa cells is in the range of 0.01–0.015 *μ*m^2^ in the experiment (see Fig 2E in Ref. [45]); correspondingly, at *t* = 10 × *τ*_BD_ ≈ 0.5 second, we get MSD ≈ 0.96 *a*^2^ ≈ 0.022 *μ*m^2^ in our simulation (see Fig 2A). Clearly, they are within the same order of magnitude. Thus, the estimate of physical time from our simulation results is sufficient for the present purpose of our study, given that the model itself is significantly coarse-grained. In comparison to the time scale estimates for chromosome dynamics in other studies [25, 29], the Brownian time *τ*_BD_, albeit a large uncertainty, is *roughly mapped* to 50 ms in physical time (*τ*_BD_ ≈ 50 ms, which is the value estimated from *η* ≈ 7 cP and monomer size *a* = 150 nm.).

Further details of the energy function and simulation algorithm are provided in the Supporting Information (S1 Text).

## Supporting information

S1 Text We provide details for (i) simulation methods, (ii) dynamics of an ideal Rouse chain, and (iii) discuss possible cause of the deviation of diffusion exponent from *β* = 0.4 in Ref. [10]. (PDF)

S1 FigProperties of the structural ensemble of Chr10 generated from our simulations.(A) Heatmap of the contact probability matrix of chromosome 10 from modeling (the upper diagonal region) and from Hi-C [9] (the lower diagonal region). For the simulated map, contact probability between monomers {*i*, *j*} was calculated as *c*_*i*,*j*_ = 〈*f*(*r*_*i*,*j*_)〉 (see Eq S6 in S1 Text). The experimental map was obtained by KR normalization [75] of the raw contact counts matrix. (B) Contact probability, $P\left(s\right)=\sum_{i=1}^{N-s}c_{i,i+s}/\left(N-s\right)$, as a function of genomic distance, *s* from our model (sim) and Hi-C (exp) [9]. (C) The average end-to-end distance with genomic separation (*s*), which obeys *R*_*ee*_(*s*) ∼ *s*^1/3^. For comparison the expected (*s*^1/2^) result for a Gaussian chain is also displayed. (D) Probability distribution of Alexander polynomial |Δ(*t*)| with *t* = −1 [47, 76, 77] calculated for the ensemble of chromosome structures generated at end of the conformational sampling. From the distribution the average number of crossings in chromosome structure is |〈Δ(−1)〉| ≈ 5.2, which allows us to estimate the average arc-length between the crossings *N*_*e*_ ≈ *Na*/〈|Δ(−1)|〉 ≈ 520*a* and thus the entanglement length of *R*_*ee*_(*s*_*e*_) ≈ 10.6*a*. (E) Normalized radial density distribution [22] of chromatin monomers with different subcompartment types, and (F) with low or high gene expression activity indicated by different RNA-seq signal levels [48]. *R*_*s*_(≈ 15*a*) is the radius of the confining sphere, and *ρ*_0_ is the average density of monomers that depends on the subcompartment type.(EPS)

S2 FigStatistical weights of *M* clusters determined from two different clustering algorithms.Statistical weights of total *M* clusters, $w\left(c\right)=\sum_{k=1}^{M}\delta (c_{k}-c)/M$, where *c*_*k*_ is the cluster index of conformation *k* determined by hierarchical clustering algorithm [78] (red) and by quality threshold algorithm [79] (blue). In hierarchical clustering, a pair of clusters with the smallest inter-cluster distance was merged together progressively, until a single cluster remains. In quality threshold algorithm, we defined the diameter of a cluster *m* as $d_{m}=\max_{k,l\in m}D_{k,l}$. The smallest cluster around every structure *k* with a diameter of *d*_*k*_ > *d*_*c*_ (= 4.5) was found. Then the largest one was removed from the ensemble. This process was repeated until all structures were clustered. Since it requires a prescribed value of the cut-off diameter *d*_*c*_, and readily leads to small clusters or singletons, hierarchical clustering method was preferred.(EPS)

S3 FigLocus-dependent diffusion.(A) Diffusion exponents and MSDs calculated for individual loci and their distribution when simulations are performed under passive condition. (left) Diffusion exponent *β* of the *i*-th locus, and the corresponding probability density for different types of loci. The exponent was calculated by fitting the time-averaged MSD of the *i*-th locus to *C* × *t*^*β*^ over the range of 10^3^ < *t*/*τ*_BD_ < 10^4^. (right) ${\bar{\text{MSD}}}_{i}$ at time *t* = 20*τ*_BD_ for the *i*-th locus, and the corresponding probability density for different types of loci (A-type in red, B-type in blue). (B) Same as (A) but the simulations were performed under active condition such that greater noises were added to the active loci. In comparison to the passive case depicted in (A), the distribution of MSD for active loci ($P({\bar{\text{MSD}}}_{i})$ in red) are clearly distinguished from that for inactive loci ($P({\bar{\text{MSD}}}_{i})$ in blue).(EPS)

S4 FigCorrelations in an ideal Rouse chain.(A) Spatial correlation of loci displacements $C_{s}^{\Delta t}\left(r\right)$ with varying lag time (Δ*t*) in an ideal Rouse chain. Symbols represent BD simulation results by using Eq 4 in the main text, and solid lines are from Equation S16 and S17 in S1 Text. (B) Correlation length *l*_*c*_ as a function of the lag time Δ*t* based on BD simulations.(EPS)

S5 FigTemporal correlation of locus dynamics.(A) ${\bar{C}}_{V,(m,m)}^{\Delta t}\left(t\right)\left[\equiv C_{V,(m,m)}^{\Delta t}\left(t\right)/C_{V,(m,m)}^{\Delta t}\left(0\right)\right]$ is a normalized mean velocity autocorrelation calculated for the midpoint monomer. The each curve represents different lag time, from Δ*t* = 100 *τ*_BD_ (dark) to 6000 *τ*_BD_ (light). (B) Correlation functions with rescaled argument, ${\bar{C}}_{V,(m,m)}^{\Delta t}\left(t/\Delta t\right)$. The theoretical curves calculated by assuming the fractional Langevin motion [60, 61] are plotted. The theoretical curve are: ${\bar{C}}_{V,(m,m)}^{\Delta t}\left(t/\Delta t\right)=({\left|t/\Delta t-1\right|}^{\beta }\;{+|t/\Delta t+1|}^{\beta }-2{\left|t/\Delta t\right|}^{\beta })/2$ with *β* = 0.4 (green) and *β* = 0.5 (white dashed line for the Rouse chain). (C) Mean velocity cross-correlation between the midpoint (*i* = *m* = *N*/2) and others (*j*), ${\bar{C}}_{V,(m,j)}^{\Delta t}\left(t\right)$ for increasing lag time Δ*t* = 125, 500, 2000, 3000 *τ*_BD_ from the top to bottom. (D) Scaling relation of Rouse modes *X*_*p*_ with *p*: $\langle X_{p}^{2}\rangle ∼p^{-\alpha }$ with *α* = 1.7 for large *p* and *α* = 1.1 for small *p*.(EPS)

S6 FigSpatial correlation of displacement vectors between two loci types.(A) Spatial correlation of displacements $C_{s}^{\Delta t}\left(r\right)$ of A-type loci (AA), B-type loci (BB), and between A-type and B-type loci (AB) with varying lag time Δ*t*. (B) Same as (A), but under active condition. The corresponding correlation lengths *l*_*c*_ as a function of lag time are plotted in Figs 4B and 6E, respectively.(EPS)

S7 FigSpatial correlation of loci displacements $C_{s}^{\Delta t}\left(r\right)$ with varying lag time (Δ*t*) in the presence of active noise.(EPS)

S8 FigReanalysis of MSND data of Zidovska et al. (Fig 4G in Ref. [10]).The fits using $\text{MSND}=A+B\times t^{\beta^{\prime }}$ (units of *A* and *B* are in *μm*^2^), which was proposed by Zidovska and coworkers, give rise to *A* = 0.00326, *B* = 0.00304, *β*′ = 0.527 for aphidicolin (cells in S phase); *A* = 0.00312, *B* = 0.00162, *β*′ = 0.584 for aphidicolin (cells NOT in S phase); *A* = 0.00350, *B* = 0.00255, *β*′ = 0.573 for *α*-amanitin; *A* = 0.00276, *B* = 0.00322, *β*′ = 0.515 for ICRF-193; *A* = 0.00187, *B* = 0.00121, *β*′ = 0.296 for ATP depleted; *A* = 0.00097, *B* = 0.00139, *β*′ = 0.846 for formaldehyde; *A* = 0.00326, *B* = 0.00304, *β*′ = 0.527 for control. On the other hand, when the data fitting is performed only over the large Δ*t* regime (Δ*t* > 4 sec), where linear relationship of MSND and Δ*t* in log-log scale is more obvious, MSND ∼ Δ*t*^*β*^ gives the scaling exponents, which are generally smaller than *β*′: *β* = 0.391 for aphidicolin (cells in S phase); *β* = 0.400 for aphidicolin (cells NOT in S phase); *β* = 0.414 for *α*-amanitin; *β* = 0.404 for ICRF-193; *β* = 0.176 for ATP depleted; *β* = 0.451 for formaldehyde; *β* = 0.490 for control. As a guide for the eye, MSND ∼ *t*^0.4^ (red line) and ∼ *t*^0.3^ (cyan line) are drawn on the graph.(EPS)

S9 Fig Comparison between the intermediate scattering functions at varying wave numbers (*k*) under passive and active conditions. When subject to active noise, *F*_*k*_(*t*) decays faster than in their absence for *k* > 2*π*/3*a*. Thus, the relaxation of Chr10 on long length scale is accelerated due to active noise.
(EPS)

S1 Movie Chromosome simulated under passive condition. (MOV)

S2 Movie Chromosome simulated under active condition. (MOV)
