# Supplementary material for: Chain organization of human interphase chromosome determines the spatiotemporal dynamics of chromatin loci
Source: PLoS Comput Biol. 2018 Dec 3;14(12):e1006617. doi: 10.1371/journal.pcbi.1006617 (PMC6292649; doi:10.1371/journal.pcbi.1006617)
Supplement: S1 Text — (PDF) [file pcbi.1006617.s001.pdf]

Supporting Information for:

Chain organization of human interphase chromosome determines the spatiotemporal dynamics of chromatin loci

Lei Liu[1], Guang Shi[2], D. Thirumalai [3], Changbong Hyeon[1*]

1 Korea Institute for Advanced Study, Seoul 02455, Korea
2 Biophysics Program, Institute for Physical Science and Technology, University of Maryland, College Park, MD 20742, USA.
3 Department of Chemistry, The University of Texas at Austin, TX 78712, USA

* hyeoncb@kias.re.kr

S1 Text

Energy function

In the coarse-grained MiChroM [1] each monomer represents 50 kb DNA that corresponds to the diameter $a = 150$ nm [2] for a single locus. For Chr10 whose length contour length is 136 Mb, the number of monomers is $N = 2712$. We used k_BT (k_B is the Boltzmann constant and T is the temperature) as the unit of energy.

Based on the distinct patterns of *inter*-chromosomal contacts and epigenetic modifications, MiChroM assigns one of the six subcompartment types $t \in \{\text{B3, B2, B1, NA, A2, A1}\}$ to each chain monomer [3]. It is found that pairs of loci, potential binding sites for CTCF [4] or lamin A [5], are in contact with higher probability than their background.

The potential in MiChroM has the form [1],

$$U_{\text{MiChroM}} = U_{\text{HP}} + \sum_{i,j} \alpha_{t_i,t_j} f(r_{ij}) + \chi \sum_{(i,j)\in\text{loops}} f(r_{ij}) + \sum_{s=3}^{s_{\max}} \gamma(s) \sum_i f(r_{i,i+s}) + \sum_{i=1}^{N} U_{\text{w}}(r_{i,w}). \quad \text{(S1)}$$

The above equation describes the energy of a homopolymer U_{HP}, monomer type (t_i, t_j)-dependent interactions, attractions between loop sites, genomic distance (s) dependent condensation energies, and repulsion due to the spherical wall.

The homopolymer term U_{HP} describes the energy of a self-avoiding chain, which we confined to a sphere with a volume fraction of $\phi = 0.1$, as

$$U_{\text{HP}} = \sum_{i=1}^{N-1} U_{\text{FENE}}(r_{i,i+1}) + \sum_{i=1}^{N-2} U_{\text{angle}}(\theta_i) + \sum_{i=1}^{N-1} U_{\text{hc}}(r_{i,i+1}) + \sum_{i=1}^{N-2}\sum_{j=i+2}^{N} U_{\text{sc}}(r_{i,j}). \quad \text{(S2)}$$

First, the neighboring monomers along the chain is constrained by the finite extensible nonlinear elastic bond potential,

$$U_{\text{FENE}}(r) = -\frac{k_b R_0^2}{2} \log\left(1 - \frac{r^2}{R_0^2}\right), \tag{S3}$$

with a spring constant $k_b = 30\ k_B T/a^2$ and a maximum extensible bond length $R_0 = 1.5a$. Second, the chain flexibility is adjusted by an angle potential,

$$U_{\text{angle}}(\theta_i) = k_a(1 - \cos(\theta_i)), \tag{S4}$$

which is defined for three consecutive monomers with $\cos\theta_i = (\hat{r}_{i,i+1} \cdot \hat{r}_{i+1,i+2})$ and $k_a = 2\ k_B T$. Third, the excluded volume interaction between neighboring monomers is modeled by using the repulsive part of the Weeks-Chandler-Andersen potential,

$$U_{\text{hc}}(r) = 4\epsilon\left[\left(\frac{a}{r}\right)^{12} - \left(\frac{a}{r}\right)^{6} + \frac{1}{4}\right]\Theta(2^{1/6}a - r), \tag{S5}$$

where $\epsilon = 1\ k_B T$ and $\Theta(\dots)$ denotes the Heaviside step function. The fourth term U_{sc} (Eq S2), which characterizes the excluded volume interaction between nonbonded monomers, has the same expression as U_{hc}. It is critical to note that during the stage of conformational sampling of chromosome structures, we have truncated the repulsive part of this potential to make $U_{sc}(r) = 2\epsilon$ at a short distance $r \leq r^*$ where $U_{\text{hc}}(r^*) = 2\epsilon$, so that chain can freely cross if necessary. This is essential for efficient conformational sampling. However, when performing BD simulations of the collapsed chromosome chain, we retain the original form of $U_{sc}(r)[= U_{hc}(r)]$, thus enforcing the excluded volume interaction.

The second to fourth terms in Eq S1 are all pairwise nonbonded attractions, which depend on the spatial distance between monomers,

$$f(r) = \frac{1}{2}\left(1 + \tanh\left[\mu(r_c - r)\right]\right), \tag{S6}$$

while the value of prefactor α_{t_i,t_j} in Eq S1 depends on the monomer types t_i and t_j.

The term with $\chi = -1.61299\ k_B T$ is for i and j contact pairs that define loops, and $\gamma(s)$ is the function of inter-loci separation $s(\leq s_{\max} = 500)$ along the chain,

$$\gamma(s) = \frac{\gamma_1}{\log(s)} + \frac{\gamma_2}{s} + \frac{\gamma_3}{s^2}. \tag{S7}$$

Lastly, the confinement effect of nuclear envelop and other chromosomes is considered as the repulsion between the wall of spherical shell (diameter of 30 a) and any monomer whose distance from the wall satisfies $r \leq 0.5a$, such that

$$U_{\text{w}}(r) = 4\epsilon\left[\left(\frac{a}{r+\Delta}\right)^{12} - \left(\frac{a}{r+\Delta}\right)^{6} + \frac{1}{4}\right]\Theta(0.5a - r). \tag{S8}$$

where $\Delta = (2^{1/6} - 0.5)a$.

All the parameters in the slightly modified MiChroM used here are summarized in Tables 1 and 2, and additional ones can be found in Ref [1].

Table 1. Parameters of the energy potential for the heteropolymer model, MiChroM.

R_0	$1.5\ a$	ϵ	$1\ k_BT$	χ	$-1.61299\ k_BT$
k_b	$30\ k_BT/a^2$	μ	3.22	γ_1	-0.03
k_a	$2\ k_BT$	r_c	$1.78\ a$	γ_2	-0.351
				γ_3	-3.727
				$s_{\max}$	500

Table 2. The monomer type dependent parameter α of MiChroM (in the unit of k_BT).

	B3	B2	B1	NA	A1	A2
B3	−0.341230	−0.329350	−0.336630	−0.349490	−0.266760	−0.301320
B2		−0.330443	−0.321726	−0.282536	−0.258880	−0.281154
B1			−0.342020	−0.209919	−0.262513	−0.286952
NA				−0.255994	−0.225646	−0.245080
A1					−0.268028	−0.274604
A2						−0.299261

Conformational sampling

To facilitate conformational sampling of the chromatin chain at equilibrium [6], underdamped Langevin equation of motion

$$m\frac{d^2\vec{r}_i}{dt^2} = -\zeta_{\mathrm{MD}}\frac{d\vec{r}_i}{dt} - \vec{\nabla}_{\vec{r}_i}U(\vec{r}_1,\vec{r}_2,\ldots) + \vec{\xi}(t) \tag{S9}$$

was integrated with a time step $\delta t = 0.01\tau_{\mathrm{MD}}$ and friction coefficient $\zeta_{\mathrm{MD}} = 0.1m/\tau_{\mathrm{MD}}$, which gives rise to the characteristic time scale of $\tau_{\mathrm{MD}} = (ma^2/\epsilon)^{1/2}$. The initial compact globular structures were obtained from an extended heteropolymer chain in the presence of heterogeneous non-bonded interaction terms for the simulation time of $2\times10^4\tau_{\mathrm{MD}}$. The truncated form of the excluded volume interaction potential was used to facilitate the conformational sampling. Then equilibration runs were performed for $10^5\tau_{\mathrm{MD}}$ for Chr10 in spherical confinement. Snapshots were collected every $10^2\tau_{\mathrm{MD}}$, from five independent replicas, for the analysis of static properties. It is worth noting that compared with the previous study [1] in which collapse for hompolymer was first induced, followed by switching on the heterogeneous non-bonded interaction terms, our procedure of obtaining the conformational ensemble by directly collapsing the heteropolymer chain is more efficient computationally; We found that the majority of resulting chromosome conformations are free from entanglement (see S1D Fig).

Simulation of chromosome dynamics

To study the dynamics (such as configurational relaxation) of the polymer chain, we carried out the simulation of chromatins under over damped condition. Because of the compact folding, hydrodynamic interactions on DNA loci will be mostly screened [7] with a marginal residual effect on the diffusivity of loci, which is supported by a recent Stokesian dynamics simulation of DNA in a packed *E. Coli* nucleoid [8]. Thus, in order to probe the dynamic behavior of chromosomes, we performed free draining Brownian dynamics (BD) simulations [9,10] by integrating the equation of motion,

$$\frac{d\vec{r}_i}{dt} = -\frac{D_{i0}}{k_BT}\nabla_{\vec{r}_i}U(\vec{r}_1,...,\vec{r}_N) + \vec{R}_i(t), \tag{S10}$$

where D_{i0} is the bare diffusion coefficient of the i-th particle, and $\vec{R}_i(t)$ is the Gaussian random noise satisfying the fluctuation-dissipation theorem

$\langle \vec{R}_i(t) \cdot \vec{R}_j(t') \rangle = 6D_{i0}\delta_{ij}\delta(t-t')$. D_{i0} was estimated via $k_BT/6\pi\eta R$, where $\eta = 7.0 \times 10^{-3}$ Pa·s is the nuclear viscosity [11] and $R = a/2$. Furthermore, since we are concerned with passive dynamics of chromosome, the truncation of the excluded volume interaction U_{sc} in Eq S2, the original intent of which was to mimic the effect of topoisomerases, was removed. Thus, in our simulation we strictly disallow chain crossing.

We chose an integration time step $\delta t_{\mathrm{BD}} = 1 \times 10^{-3}\ \tau_{\mathrm{BD}}$ with the Brownian time $\tau_{\mathrm{BD}} = a^2/D_{i0} \approx 50$ ms, which is the value estimated from $\eta \approx 7$ cP and monomer size $a = 150$ nm. Starting from centroid conformations of the five most populated clusters (Fig 1B), BD simulations were carried out for $4 \times 10^4\ \tau_{\mathrm{BD}}$ in each trajectory. Unless stated otherwise, all the time scales of dynamic quantities reported in this study are in the unit of τ_{BD}, and the genomic position are measured in the unit of Mb. All simulations were performed by adapting the ESPResSo 3.3.1 package [12, 13].

Correlation in time: Velocity-velocity auto-correlation

We calculated the correlation function of displacement of the ith and j^{th} loci divided by the lag time Δt, which is equivalent to the mean velocity correlation function [14, 15],

$$C^{\Delta t}_{V,(i,j)}(t) = \frac{\langle \Delta \vec{r}_i(t+t_0;\Delta t) \cdot \Delta \vec{r}_j(t_0;\Delta t)\rangle_{t_0}}{(\Delta t)^2}, \quad \text{(S11)}$$

where $\Delta \vec{r}_i(t;\Delta t) = \vec{r}_i(t+\Delta t) - \vec{r}_i(t)$ and $\langle \ldots \rangle_{t_0} \equiv 1/\tau_{\max}\int_0^{\tau_{\max}} dt_0(\ldots)$. Regardless of Δt, the auto-correlation function $C^{\Delta t}_{V,(m,m)}(t)$, calculated for the midpoint monomer ($m = N/2$), displays a negative correlation peak ($C^{\Delta t}_{V,(m,m)} < 0$) at $t = \Delta t$ (S5A Fig), followed by a slow relaxation to zero, i.e., $C^{\Delta t}_{V,(m,m)}(t \gg \Delta t) \to 0$. The curves plotted with the rescaled time $t/\Delta t$ overlap onto each other and allow us to assess the variation among the curves (S5B Fig).

Following the interpretation of fractional Langevin motion, one could posit that the dynamic behavior of chromatin loci captured in $C^{\Delta t}_{V,(m,m)}(t)$ is caused by viscoelasticity of the effective medium [16]. However, even an ideal Rouse chain in free space ($\beta = 0.5$) displays a similar curve $C^{\Delta t}_{V,(m,m)}(t)$ (S5B Fig, white dashed line). The negative correlation peak for the Rouse chain is solely due to the chain connectivity with the neighboring monomer along the chain. For our chromosome model, restoring forces of the surrounding, non-covalently interacting beads can contribute to the negative correlation peak as well. As shown in S5B Fig, the difference between the two curves, $C^{\Delta t}_{V,(m,m)}(t/\Delta t)$s, with $\beta = 0.4$ for our chromatin model and with $\beta = 0.5$ for the ideal Rouse chain is subtle, and is not easy to discern.

The dynamical behavior of our chromatin model can more straightforwardly be discerned from that of the ideal Rouse chain by calculating the Rouse modes, $\vec{X}_p(t) = N^{-1}\sum_{n=1}^{N}\cos(pn\pi/N)\vec{r}_n(t)$. While $\langle X_p^2\rangle \sim p^{-2}$ is anticipated for the free Rouse chain [7, 17], we find $\langle X_p^2 \rangle \sim p^{-1.7}$ for large p ($N/p \lesssim 100$. See S5D Fig). The Rouse modes for a chain with the exponent ν are expected [18] to scale as $\langle X_p^2\rangle \sim p^{-(1+2\nu)}$. Thus, the exponent of 1.7 is explained again by the SF statistics with $\nu = 1/3$.

Cross-correlations of mean velocity between the midpoint ($m = N/2$) and other loci ($j \neq N/2$) show how the correlation of our chromatin model changes with time (S5C Fig). In contrast to the viscoelastic Rouse polymer model [19], the mean velocity cross-correlation reveals a non-uniform and undiminishing correlation pattern, which suggests that the chromosome structure is maintained through heterogeneous loci interactions defying a full equilibration.

Displacement correlation in an ideal Rouse chain

The coordinates of the ith monomer $\vec{r}_i$ in an ideal Rouse chain can be transformed into a linear combinations of different Rouse modes $\vec{X}_p$ [7],

$$\vec{r}_i(t) = \vec{X}_0(t) + 2\sum_{p=1}^{N-1} \vec{X}_p(t)\cos\left(\frac{p\pi i}{N}\right). \tag{S12}$$

Then, the position correlation between the ith and j^{th} monomers, at time $t+\Delta t$ and t respectively, can be written as

$$\begin{aligned}\vec{r}_i(t+\Delta t)\vec{r}_j(t) &= \vec{X}_0(t+\Delta t)\vec{X}_0(t)\\ &+ 4\sum_{p=1}^{N-1}\sum_{q=1}^{N-1} \vec{X}_p(t+\Delta t)\vec{X}_q(t)\cos\left(\frac{p\pi i}{N}\right)\cos\left(\frac{q\pi j}{N}\right).\end{aligned} \tag{S13}$$

Given that

$$\langle X_{p,\alpha}(t)X_{q,\beta}(t')\rangle = \delta_{pq}\delta_{\alpha\beta}\frac{k_BT}{k_p}\exp\left(-\frac{|t'-t||}{\tau_p}\right) \tag{S14}$$

for $p > 0$ and $\langle X_{0,\alpha}(t)X_{0,\beta}(t')\rangle = \delta_{\alpha\beta}2k_BT/\zeta N \times \min(t,t')$, the average position correlation in 3D is

$$\langle \vec{r}_i(t+\Delta t)\vec{r}_j(t)\rangle = \frac{6k_BT}{N\zeta}t + 12\sum_{p=1}^{N-1}\frac{k_BT}{k_p}e^{-\Delta t/\tau_p}\cos\left(\frac{p\pi i}{N}\right)\cos\left(\frac{q\pi j}{N}\right), \tag{S15}$$

where $k_p = 6\pi^2p^2k_BT/Nb^2$ and $\tau_p = \zeta N^2b^2/3\pi^2p^2k_BT$ for $p > 0$. Consequently, the average displacement correlation between the ith and j^{th} monomers, at waiting time Δt, will be

$$\begin{aligned}C_{i,j}(\Delta t) &= \langle\Delta\vec{r}_i(t;\Delta t)\Delta\vec{r}_j(t;\Delta t)\rangle\\ &= \langle\vec{r}_i(t+\Delta t)\vec{r}_j(t+\Delta t)\rangle + \langle\vec{r}_i(t)\vec{r}_j(t)\rangle - 2\langle\vec{r}_i(t+\Delta t)\vec{r}_j(t)\rangle\\ &= \frac{6k_BT}{N\zeta}\Delta t + 24\sum_{p=1}^{N-1}\frac{k_BT}{k_p}\left[1-e^{-\frac{\Delta t}{\tau_p}}\right]\cos\left(\frac{p\pi i}{N}\right)\cos\left(\frac{q\pi j}{N}\right).\end{aligned} \tag{S16}$$

The first term in the last row of Eq S16 increases linearly with the lag time Δt, which is contributed from the zeroth mode $\vec{X}_0$ describing diffusion of the center of mass. The second term contains contributions from other modes $\vec{X}_{p(>0)}$, which varies as $1-\exp(-\Delta t/\tau_p)$ and saturates to a constant ($\propto \sum_{p=1}^{N-1}\cos(p\pi i/N)\cos(q\pi j/N)$) as $t\to\infty$. Due to the lack of the genomic positions of chromatin loci (i.e., the values of i and j) in the experiment [20], we calculated the spatial correlation function $C_s^{\Delta t}(r)$ (defined in Eq 4 in the main text) as

$$C_s^{\Delta t}(r) \approx \frac{\sum_{i>j} C_{i,j}(\Delta t)P_{i,j}(r)}{\sum_{i>j} P_{i,j}(r)} \tag{S17}$$

which calibrates the displacement correlation between two loci separated by the distance r over the time interval Δt. $P_{i,j}(r)$ is the probability density function of the distance r between the ith and j^{th} monomers, which follows [7]

$$P_{i,j}(r) = 4\pi r^2\left(\frac{3}{2\pi b^2|i-j|}\right)^{3/2} e^{-\frac{3r^2}{2b^2|i-j|}}. \tag{S18}$$

Eq S16 and S17 are compared with additional simulations of an ideal chain composed of $N = 100$ monomers, where neighboring monomers along the chain are constrained by harmonic interactions [14]

$$U = \sum_{i=1}^{N-1} \frac{3k_BT}{2b^2}(\vec{r}_{i+1} - \vec{r}_i)^2. \tag{S19}$$

BD simulations were carried out by integrating Eq S10 with $k_BT = 1$, $\zeta = 1$, and $b = 0.5477$.

Given that the correlation length l_c for an ideal Rouse chain increases with lag time Δt (S4 Fig), the coherent motion of chromatin loci observed at large Δt itself should not be too surprising (see Fig 4). In the presence of confinement of size R_s, the diffusion of the whole chain would eventually be constrained by the confinement; the first term in the displacement correlation (Eq S16) for the Rouse chain would saturate to R_s^2 at long time limit, and the confinement effect on the intrachain motion would be negligible as long as $R_s^2 > \langle R_g^2 \rangle = Nb^2/6$. As clearly demonstrated in S4 Fig, the displacement correlation increases *monotonically* for ideal Rouse chain.

It is important to note that there is a reduction in l_c at large Δt (*non-monotonic* change of l_c with Δt) in live cells [20] as well as in our simulations when isotropic active noise is included (Fig 6D).

Exact result for $\delta(t)$ for an ideal Rouse chain

It is instructive to compute $\delta(t)$,

$$\delta(t) = \sqrt{\frac{2}{N(N-1)} \sum_{i<j} (r_{ij}(t) - r_{ij}(0))^2} \tag{S20}$$

for an ideal Rouse polymer because the value of $\lim_{t\to\infty}\langle\delta(t)\rangle = \delta_{\text{eq}}$ obtained for this model is an upper bound for any chromosome. Based on Eq S12, the vector between the ith and j^{th} monomers is,

$$\begin{aligned} \vec{R}_{ij}(t) &= \vec{r}_i(t) - \vec{r}_j(t) \\ &= 2\sum_{p=1}^{N-1} \vec{X}_p(t) \left[\cos\left(\frac{p\pi i}{N}\right) - \cos\left(\frac{p\pi j}{N}\right)\right]. \end{aligned} \tag{S21}$$

The Rouse normal mode, $\vec{X}_p(t)$, is the solution of the Langevin equation

$$\zeta_p \frac{\partial}{\partial t}\vec{X}_p = -k_p\vec{X}_p(t) + \vec{f}_p(t) \tag{S22}$$

where $\zeta_0 = N\zeta$ and $\zeta_p = 2N\zeta$ for $p > 0$, $k_p = \frac{6\pi^2 k_BT}{Na^2}p^2 \equiv \omega p^2$ and $\langle f_{p\alpha}(t) f_{p\beta}(t')\rangle = 2\delta_{pq}\delta_{\alpha\beta}\zeta_p k_BT\delta(t-t')$.

Let us denote $\phi_{ij}(t) \equiv \langle(|\vec{R}_{ij}(t)| - |\vec{R}_{ij}(0)|)^2\rangle$, where $\langle\cdots\rangle$ is the ensemble average over both initial condition and the conformations. We use $\overline{x}$ and $\mathbf{E}[x]$ to label average over conformations and average over the initial condition, respectively. With this notation, $\phi_{ij}(t) = \mathbf{E}[\overline{R_{ij}^2(t)}] + \mathbf{E}[R_{ij}^2(0)] - 2\mathbf{E}[|R_{ij}(0)|\overline{|R_{ij}(t)|}]$. The function $X_{p,\alpha}$ (α is one of the three components of $\vec{X}_p(t)$) is a normal random variable with mean $X_{p,\alpha}(0)e^{-t/\tau_p}$ and variance $(k_BT/k_p)(1 - e^{-2t/\tau_p})$, which we write as

$$X_{p,\alpha}(t) \sim \mathcal{N}\big(X_{p,\alpha}(0)e^{-t/\tau_p}, \frac{k_BT}{k_p}(1 - e^{-2t/\tau_p})\big). \tag{S23}$$

In the time limit, $t \to \infty$, the distribution of $R_{ij}(t)$ does not depend on the initial condition (system is ergodic). Therefore, we obtain $\lim_{t\to\infty} X_{p,\alpha}(t) \sim \mathcal{N}\big(0, \frac{k_BT}{k_p}\big)$. Because $R_{ij,\alpha}(t)$ is linear combination of $X_{p,\alpha}$ it follows that $R_{ij,\alpha}(t)$ is also a normal random variable. Consequently,

$$\begin{aligned}&\lim_{t\to\infty} R_{ij,\alpha}(t)\\ &\sim \mathcal{N}\left(0, 4\sum_{p=1}^{N-1}\left[\cos\left(\frac{p\pi i}{N}\right) - \cos\left(\frac{p\pi j}{N}\right)\right]^2 \frac{k_BT}{k_p}\right).\end{aligned} \tag{S24}$$

Since $R_{ij}^2(t) = \sum_\alpha R_{ij,\alpha}^2(t)$, then $\lim_{t\to\infty} R_{ij}^2(t)$ is a non-central chi-squared random variable. Denoting $\sigma_{ij,x}^2 = \sigma_{ij,y}^2 = \sigma_{ij,z}^2 = \sigma_{ij}^2 \equiv 4\sum_{p=1}^{N-1}[\cos(\frac{p\pi i}{N}) - \cos(\frac{p\pi j}{N})]^2 \frac{k_BT}{k_p}$, we obtain $\mathbf{E}[\overline{R_{ij}^2(t)}] = \sum_{\alpha=x,y,z}\mathbf{E}[\overline{R_{ij,\alpha}^2(t)}] = 3\sigma_{ij}^2$. Since the distribution of $R_{ij,\alpha}(t)$ in the long time limit is the equilibrium distribution, we have $\mathbf{E}[R_{ij}^2(0)] = \mathbf{E}[\overline{R_{ij}^2(t)}] = 3\sigma_{ij}^2$.

In order to calculate $\mathbf{E}[|R_{ij}(0)|\overline{|R_{ij}(t)|}]$ we note that $\overline{|R_{ij}(t)|}$ is independent of the initial condition. Thus, $\lim_{t\to\infty}\mathbf{E}[|R_{ij}(0)|\overline{|R_{ij}(t)|}] = \mathbf{E}[|R_{ij}(0)|]\lim_{t\to\infty}\overline{|R_{ij}(t)|} = (\lim_{t\to\infty}\overline{|R_{ij}(t)|})^2$. It can be shown that $\lim_{t\to\infty}|R_{ij}(t)| = \lim_{t\to\infty}\sqrt{\sum_\alpha R_{ij,\alpha}^2(t)}$. Combining the results for $\mathbf{E}[\overline{R_{ij}^2(t)}]$ and $\mathbf{E}[|R_{ij}(0)|\overline{|R_{ij}(t)|}]$ we obtain,

$$\begin{aligned}\lim_{t\to\infty}\phi_{ij}(t) &= \mathbf{E}[\overline{R_{ij}^2(t)}] + \mathbf{E}[R_{ij}^2(0)] - 2\mathbf{E}[|R_{ij}(0)|\overline{|R_{ij}(t)|}]\\ &= \left(6 - \frac{16}{\pi}\right)\sigma_{ij}^2 \equiv c\sigma_{ij}^2.\end{aligned} \tag{S25}$$

The value of $\Lambda_{eq} = \lim_{t\to\infty}\delta(t)^2$ in the long time limit is,

$$\Lambda_{eq} = \frac{2}{N(N-1)}\sum_{i<j}\lim_{t\to\infty}\phi_{ij}(t) = \frac{2c}{N(N-1)}\sum_{i<j}\sigma_{ij}^2. \tag{S26}$$

The quantity $\sum_{i<j}\sigma_{ij}^2$ is evaluated as follows,

$$\begin{aligned}\sum_{i<j}\sigma_{ij}^2 &= \frac{4k_BT}{\omega}\sum_{i<j}\sum_{p=1}^{N-1}\left[\cos\left(\frac{p\pi i}{N}\right) - \cos\left(\frac{p\pi j}{N}\right)\right]^2\frac{1}{p^2}\\ &\approx \frac{4k_BT}{\omega}\sum_{i<j}\frac{5|i-j|}{N} = \frac{20k_BT}{\omega N}\sum_{s=1}^{N-1}(N-s)s\\ &= \frac{10k_BT}{3\omega}(N-1)(N+1).\end{aligned} \tag{S27}$$

Substituting Eq S27 in Eq S26 we obtain,

$$\begin{aligned}\Lambda_{eq} &= \frac{2c}{N(N-1)}\frac{10k_BT}{3\omega}(N-1)(N+1)\\ &= \frac{20ck_BT}{3\omega}\frac{N+1}{N} = \frac{10}{9\pi^2}\left(6 - \frac{16}{\pi}\right)a^2(N+1)\\ &\approx 0.1a^2N.\end{aligned} \tag{S28}$$

The equilibrium value, δ_{eq}, is given by $\sqrt{\Lambda_{eq}}$. For $N = 2712$ we obtain $\delta_{eq} \approx 16.5a$, which is the upper bound of the excursion of any two loci in chromosomes.

Deviation of diffusion exponent from $\beta = 0.4$

As discussed in the main text our finding that the growth of the mean square displacement, $\mathrm{MSD}(t) \approx t^{\beta}$ with $\beta = 0.4$ is consistent with a number of experiments. A notable exception appears to be in the experimental study of Zidovska *et al.* [20] who found that a quantity $\mathrm{MSND}(t)$ obtained from displacement correlation spectroscopy, which is not unrelated to MSD, depends on ATP. In the presence of ATP, presumably the analog of the simulations in the presence of active forces, the value of $\beta \approx 0.71$ for the loci motion averaged over the whole genome in 16 nuclei. However, when ATP is depleted β decreases to ≈ 0.32. Superficially, it might appear that our results are not consistent with their findings [20]. However, there might be genuine differences, which might make it difficult to compare the experimental and simulation results. A few technical points are worth noting.

1. In MSND (mean square network displacement) analysis, which is likely related to MSD, Zidovska and colleagues used the following relationship to fit the data over the entire range of time span Δt: $\mathrm{MSND} = A + B \times \Delta t^{\beta'}$, where A (whose value ought to be zero in any fit to MSD) and B are constants. The parameters A, B, and β' obtained from such a fit over the time regime tend to overestimate the value of β from $\mathrm{MSD} \sim \Delta t^{\beta}$ at large Δt, which is more generally used in other studies [21–24]. We reanalyzed the the time dependence of the MSND to extract the β values, and found that data are fitted with $\beta \approx 0.5$ for the "control," $\beta \approx 0.4$ for many different types of drug-treated cells, and $\beta \approx 0.18$ for the ATP-depleted cell (see S8 Fig for details).

2. Both β' and β values for ATP depleted cell discussed above are significantly smaller than the diffusion exponent 0.4. The images of live cells and ATP-depleted cells are *visually different* (compare Fig 4A and Fig 4B in Ref. [20]. In addition, according to other independent measurements, reporting ATP-level dependent compaction and the recovery of original cell state after the "wash" [25, 26], ATP depletion induces chromatin compaction and substantially slows down of the dynamics via a caging effect [27, 28], as found in glassy systems. We surmise that to experimentally acquire the desired diffusion exponent $\beta \approx 0.4 - 0.5$ from ATP-depleted condition that suppresses the biological activity, it is necessary that the lag time (Δt) for MSD measurement be greater than the time scale associated with caging. In other words the scarcity of data points beyond $\Delta t \approx 2s$ (S8 Fig) and the restriction that the maximum value of Δt to $\approx$ 10s makes it difficult to conclude definitively that ATP-depletion significantly change the exponent of the time-dependence of MSND. Measurements for much longer Δt values are needed to compare with our simulations and other experimental studies. A study on bacterial chromosome showed clearly that the β exponent remains almost identical to be $\beta = 0.4$ even when the ATP is depleted, but that the diffusivity is reduced significantly [21, 22]

References

1. Di Pierro M, Zhang B, Aiden EL, Wolynes PG, Onuchic JN. Transferable model for chromosome architecture. Proc Natl Acad Sci USA. 2016;113(43):12168–12173.

2. Zhang B, Wolynes PG. Topology, structures, and energy landscapes of human chromosomes. Proc Natl Acad Sci USA. 2015;112(19):6062–6067.

3. Rao SSP, Huntley MH, Durand NC, Stamenova EK, Bochkov ID, Robinson JT, et al. A 3D Map of the Human Genome at Kilobase Resolution Reveals Principles of Chromatin Looping. Cell. 2014;159(7):1665–1680.
4. Sanborn AL, Rao SSP, Huang SC, Durand NC, Huntley MH, Jewett AI, et al. Chromatin extrusion explains key features of loop and domain formation in wild-type and engineered genomes. Proc Natl Acad Sci USA. 2015;112(47):E6456–E6465.
5. Bronshtein I, Kepten E, Kanter I, Berezin S, Lindner M, Redwood AB, et al. Loss of lamin A function increases chromatin dynamics in the nuclear interior. Nat Commun. 2015;6:8044.
6. Honeycutt JD, Thirumalai D. The nature of folded states of globular proteins. Biopolymers. 1992;32(6):695–709.
7. Doi M, Edwards SF. The Theory of Polymer Dynamics (International Series of Monographs on Physics). Oxford University Press; 1988.
8. Chow E, Skolnick J. DNA Internal Motion Likely Accelerates Protein Target Search in a Packed Nucleoid. Biophys J. 2017;112(11):2261–2270.
9. Hyeon C, Thirumalai D. Multiple Probes are Required to Explore and Control the Rugged Energy Landscape of RNA Hairpins. J Am Chem Soc. 2008;130(5):1538–1539.
10. Hinczewski M, von Hansen Y, Dzubiella J, Netz RR. How the diffusivity profile reduces the arbitrariness of protein folding free energies. J Chem Phys. 2010;132(24):245103.
11. Hajjoul H, Mathon J, Ranchon H, Goiffon I, Mozziconacci J, Albert B, et al. High-throughput chromatin motion tracking in living yeast reveals the flexibility of the fiber throughout the genome. Genome Res. 2013;23(11):1829–1838.
12. Limbach HJ, Arnold A, Mann BA, Holm C. ESPResSo – An Extensible Simulation Package for Research on Soft Matter Systems. Comput Phys Commun. 2006;174(9):704–727.
13. Arnold A, Lenz O, Kesselheim S, Weeber R, Fahrenberger F, Roehm D, et al. ESPResSo 3.1 — Molecular Dynamics Software for Coarse-Grained Models. In: Griebel M, Schweitzer MA, editors. Meshfree Methods for Partial Differential Equations VI. vol. 89 of Lecture Notes in Computational Science and Engineering. Springer; 2013. p. 1–23.
14. Weber SC, Theriot JA, Spakowitz AJ. Subdiffusive motion of a polymer composed of subdiffusive monomers. Phys Rev E. 2010;82:011913.
15. Lucas JS, Zhang Y, Dudko OK, Murre C. 3D Trajectories Adopted by Coding and Regulatory DNA Elements: First-Passage Times for Genomic Interactions. Cell. 2014;158(2):339–352.
16. Panja D. Anomalous polymer dynamics is non-Markovian: memory effects and the generalized Langevin equation formulation. J Stat Mech Theory E. 2010;2010(06):P06011.
17. Hsu HP, Kremer K. Detailed analysis of Rouse mode and dynamic scattering function of highly entangled polymer melts in equilibrium. Eur Phys J Spec Top. 2017;226(4):693–703.

18. Shinkai S, Nozaki T, Maeshima K, Togashi Y. Dynamic Nucleosome Movement Provides Structural Information of Topological Chromatin Domains in Living Human Cells. PLoS Comput Biol. 2016;12(10):1–16.

19. Lampo TJ, Kennard AS, Spakowitz AJ. Physical Modeling of Dynamic Coupling between Chromosomal Loci. Biophys J. 2016;110(2):338–347.

20. Zidovska A, Weitz DA, Mitchison TJ. Micron-scale coherence in interphase chromatin dynamics. Proc Natl Acad Sci USA. 2013;110(39):15555–15560.

21. Weber SC, Spakowitz AJ, Theriot JA. Bacterial chromosomal loci move subdiffusively through a viscoelastic cytoplasm. Phys Rev Lett. 2010;104(23):238102.

22. Weber SC, Spakowitz AJ, Theriot JA. Nonthermal ATP-dependent fluctuations contribute to the in vivo motion of chromosomal loci. Proc Natl Acad Sci U S A. 2012;109(19):7338–7343.

23. Bronstein I, Israel Y, Kepten E, Mai S, Shav-Tal Y, Barkai E, et al. Transient anomalous diffusion of telomeres in the nucleus of mammalian cells. Phys Rev Lett. 2009;103(1):018102.

24. Jeon JH, Monne HMS, Javanainen M, Metzler R. Anomalous diffusion of phospholipids and cholesterols in a lipid bilayer and its origins. Phys Rev Lett. 2012;109(18):188103.

25. Llères D, James J, Swift S, Norman DG, Lamond AI. Quantitative analysis of chromatin compaction in living cells using FLIM-FRET. J Cell Biol. 2009;187(4):481–496.

26. Visvanathan A, Ahmed K, Even-Faitelson L, Lleres D, Bazett-Jones DP, Lamond AI. Modulation of higher order chromatin conformation in mammalian cell nuclei can be mediated by polyamines and divalent cations. PLoS One. 2013;8(6):e67689.

27. Hameed FM, Rao M, Shivashankar G. Dynamics of passive and active particles in the cell nucleus. PLoS One. 2012;7(10):e45843.

28. Liu J, Vidi PA, Lelièvre SA, Irudayaraj JM. Nanoscale histone localization in live cells reveals reduced chromatin mobility in response to DNA damage. J Cell Sci. 2015;128(3):599–604.
